# Current Trends in the Imaging Diagnosis of Neonatal Respiratory Distress Syndrome (NRDS): Chest X-ray Versus Lung Ultrasound

**DOI:** 10.7759/cureus.69787

**Published:** 2024-09-20

**Authors:** Alexandra E Popa, Simona D Popescu, Adriana Tecuci, Mihaela Bot, Simona Vladareanu

**Affiliations:** 1 Obstetrics and Gynaecology and Neonatology, Elias Emergency University Hospital, Bucharest, ROU; 2 Obstetrics and Gynaecology and Neonatology, Carol Davila University of Medicine and Pharmacy, Bucharest, ROU; 3 Obstetrics and Gynaecology, Elias Emergency University Hospital, Bucharest, ROU; 4 Obstetrics and Gynaecology, Carol Davila University of Medicine and Pharmacy, Bucharest, ROU

**Keywords:** chest x-ray, diagnosis, lung ultrasound, neonatal intensive care unit, neonatal respiratory distress syndrome

## Abstract

Neonatal respiratory distress syndrome (NRDS) is a major cause of morbidity and mortality in newborns, particularly in neonatal intensive care units (NICUs). Until recently, its diagnosis had been based on clinical signs, arterial blood gas analysis, and chest X-ray (CXR). However, the frequent use of CXR exposes newborns to ionizing radiation, which can have long-term negative effects, including an increased risk of cancer, especially among premature infants. Lung ultrasound (LUS) has been proposed as a promising alternative for diagnosing NRDS due to its many advantages: no exposure to radiation, the ability to be performed at the bedside, repeatability, and ease of use.

This review compared the diagnostic accuracy of LUS with the reference standard, CXR, in evaluating NRDS in newborns admitted to the NICU. Studies have shown that LUS can identify specific signs of NRDS, such as bilateral "white lung," pleural line abnormalities, and lung consolidations. The method has high sensitivity and specificity for diagnosing this condition and offers several advantages over other diagnostic methods; it does not involve ionizing radiation, thereby eliminating the risk of radiation exposure; it is cost-effective, easy to use, and can be performed at the patient's bedside, making it a viable alternative to CXR for reducing ionizing radiation exposure. Additionally, LUS can be used to monitor the progression of respiratory diseases and guide clinical management, especially in determining the optimal timing for surfactant administration in newborns with respiratory distress syndrome (RDS).

We conclude that LUS is an effective and non-invasive alternative method for diagnosing and managing NRDS, with the potential to improve the safety and quality of care in the NICU, where rapid and safe diagnostic tools are essential for managing the health of newborns.

## Introduction and background

Respiratory pathologies in newborns are the most common reason for admission to the neonatal intensive care unit (NICU). Moreover, they are the leading cause of early morbidity and mortality (0-7 days) in newborns [1]. Clinical signs and simple chest X-rays (CXR) are key in diagnosing neonatal respiratory diseases. However, these methods can sometimes present diagnostic challenges for neonatologists due to the low sensitivity and specificity of signs and symptoms, which may not always be resolved with CXRs alone. Consequently, the diagnosis may be imprecise, potentially leading to delayed management of acute conditions. Neonatal ultrasound was first described in the 1960s, and it gained wide currency in the last decade. The guidelines of the European Society of Paediatric and Neonatal Intensive Care (ESPNIC) on the use of point-of-care ultrasound (POCUS) in newborns highlight the importance of LUS in assessing critically ill patients in NICUs [1].

The use of lung ultrasound (LUS) over the past decade as an alternative to CXR for diagnosing neonatal lung diseases has been described in several studies. In newborns, LUS imaging can be very useful, benefiting from the neonatal anatomical characteristics of having a small chest width and lung mass with a thin chest wall. Although it is performed indirectly, LUS allows for a satisfactory visualization of the lungs. One of the most significant benefits of LUS is that it is a radiation-free method. It is relatively easy for the examiner to learn and is technically less demanding than other examinations [2,3].

CXR is the primary method used to evaluate neonatal respiratory distress syndrome (NRDS) in newborns. However, frequent exposure to ionizing radiation can have long-term adverse effects, including an increased risk of cancer, especially in premature infants. Research suggests that LUS may be a useful alternative for diagnosing NRDS. Reducing the dose of ionizing radiation is one of the main objectives of pediatric radiology. Thus, the continuous search for a balance between potential benefits and delayed adverse effects, which may result from using radiation-based diagnostic procedures, is critical when working with newborns.

In clinical practice, newborns are often subjected to repeat CXRs, used for the initial diagnosis of conditions such as respiratory distress syndrome (RDS), pneumonia, pneumothorax, meconium aspiration, congenital heart diseases, pulmonary malformations, and lung masses. These X-rays also aid in monitoring disease progression, evaluating therapy effectiveness, and checking the position of endotracheal tubes and central venous catheters. Each exposure contributes to the cumulative increase in ionizing radiation, and while the dose received with each exposure is low, it is essential to minimize these exposures, especially in newborns and premature infants, who are particularly vulnerable to the adverse effects of radiation. In this patient population, the risk of cancer associated with a given dose of radiation is considered to be two to three times higher than in the general population and six to nine times higher than in 60-year-olds. Therefore, the diagnosis and monitoring of respiratory diseases in newborns should be performed using alternative methods not associated with these potentially harmful effects [4].

In this study, we aimed to compare the sensitivity and specificity of LUS versus chest CXR in diagnosing NRDS and other respiratory conditions in newborns. This includes analyzing pooled data from multiple studies to provide a robust comparison of the two imaging modalities. We also sought to assess the reduction in neonatal exposure to ionizing radiation by using LUS instead of CXR in NICUs. This objective would involve quantifying the potential decrease in radiation doses received by newborns and evaluating the implications for long-term health outcomes, particularly concerning cancer risk and DNA damage. Also, we endeavored to evaluate the advantages of LUS over CXR in providing real-time monitoring of lung conditions in neonates. This involves studying how LUS can be used dynamically at the bedside to track changes in lung pathology and adjust treatments promptly. More research is required to understand the learning curve associated with LUS and to determine the most effective training programs for healthcare professionals.

## Review

Neonatal respiratory distress syndrome

Previously described as hyaline membrane disease, NRDS is a condition specific to premature newborns. It is caused by an insufficient amount of surfactant in the pulmonary alveoli. This insufficiency leads to alveolar collapse, pulmonary instability at the end of expiration, reduced lung volume, decreased compliance, and impaired gas exchange [5].

Pathophysiology

The primary cause of NRDS is prematurity and pulmonary immaturity, but there can also be genetic mutations in the genes encoding surfactant proteins or defects in an ATP transporter protein, leading to a hereditary condition in full-term newborns [6]. The surfactant deficiency can result in pulmonary inflammation and epithelial injury, increasing pulmonary resistance. Additionally, abnormal absorption of pulmonary fluid leads to inefficient clearance, causing pulmonary edema and impaired gas exchange [6].

The treatment of RDS has significantly improved since the advent of surfactant therapy. It has been observed that the administration of antenatal corticosteroids, the use of positive end-expiratory pressure to prevent atelectasis, and exogenous surfactant therapy have significantly improved the course of the disease and reduced the severity of RDS [7]. Differential diagnosis can often be made using two imaging methods, especially for differentiating respiratory pathologies such as transient neonatal tachypnea, congenital pneumonia, pneumothorax, and cardiac pathologies like cyanotic heart diseases [8,9,10].

Lung ultrasound in neonatal respiratory distress syndrome

Neonatal LUS can be used in emergencies to differentiate neonatal respiratory pathologies, thereby reducing neonatal morbidity [11]. LUS has the advantage of being performed at the newborn’s bedside, allowing for dynamic repetition without exposing the infant to ionizing radiation. Its functional and descriptive applications make LUS a high-fidelity tool for aiding in the differential diagnosis of neonatal respiratory failure causes and for guiding subsequent management [11,12].

Compared to CXR, LUS has higher sensitivity and specificity for diagnosing respiratory pathologies (e.g., pneumothorax and pleural effusions) and can be used for clinical monitoring of these conditions. LUS can be performed by neonatologists and offers the significant advantage of immediate interpretation by caregivers, helping to establish an accurate diagnosis and enabling timely therapeutic intervention. Currently, the widespread use of POCUS is limited by the lack of a standardized educational program and the absence of effective quality assurance programs, although it has multiple applications and benefits [11,12].

In neonatal ultrasound, a high-frequency linear probe, usually greater than 10 MHz, is most frequently used. This probe offers the advantage of high resolution with a shallow penetration depth. Higher frequency probes, such as hockey-stick-shaped microlinear probes, are commonly used for vascular access but can also be used in premature newborns due to their small footprint. Moreover, various types of probes can be utilized by an experienced neonatologist to recognize pathology through LUS. The most common modes used in LUS are B-mode (brightness) and M-mode (motion), along with the use of color Doppler to evaluate blood flow. Neonatal LUS is usually performed in the supine position, allowing direct visualization of the subcutaneous tissue, ribs, and pleural line, and indirect evaluation of lung tissue [11,12].

LUS is particularly useful in neonatology due to the neonatal chest anatomy, unique chest ossification, and respiratory pathologies. The unique anatomy of the pediatric chest, including its unossified thorax, large thymus, and thin subcutaneous tissue, provides excellent acoustic windows for LUS, allowing for effective imaging of the chest, mediastinum, and pleura, though ossification with age reduces this accessibility [13,14].

Ultrasound can create sections in all planes but requires examiner experience and a good understanding of topographical anatomy and organ exploration methods [15]. All ultrasound diagnostic methods are based on the principle that ultrasound waves are reflected by an interface between media with different acoustic impedances [16]. In normal air-filled lungs, ultrasound is limited because there is no acoustic mismatch in the ultrasound beam when it encounters air [17]. The pleural line and repetitive horizontal hyperechoic A-lines can be visualized by ultrasound. The pleural line is a smooth, regular, hyperechoic line that moves with respiration [18]. A-lines are parallel lines located at regular intervals below the pleural line and represent the change in acoustic impedance at the pleura-lung interface, generating horizontal artifacts. B-lines appear when air content decreases, and subpleural interstitial edema forms; these are hyperechoic images that originate from the pleural line and move with respiration [19].

B-lines correlate with the pulmonary interstitial fluid content, and their number increases as air content decreases. Multiple B-lines indicate an alveolar-interstitial syndrome. The presence of compact B-lines in the lung fields implies a severe alveolar-interstitial syndrome, known as "white lung." When air content decreases further, the lung parenchyma becomes visible by opening an acoustic window on the lung [17]. Lung consolidation is described as a region with poorly defined or wedge-shaped hypoechoic margins. The presence of air bronchograms or vascular patterns can help identify the etiology of lung consolidation [20].

The main ultrasound features of NRDS include lung consolidations, the presence of air bronchograms, pleural line abnormalities, pleural effusions, and the bilateral white lung appearance. Among these, lung consolidation is the most significant indicator of NRDS, useful for both establishing the diagnosis and staging the severity of the condition. Additionally, LUS has proven valuable in diagnosing complications that can arise from this syndrome, such as pulmonary hemorrhage, pneumothorax, or atelectasis [12,13]. Another role of LUS is to assist in differential diagnosis, particularly between neonatal RDS and transient tachypnea of the newborn (TTN). While RDS and TTN often present with similar early clinical symptoms, making them difficult to distinguish, LUS can help differentiate the two conditions. RDS is characterized by subpleural lung consolidation, a key ultrasound feature, whereas TTN typically involves pulmonary edema without any lung consolidation. Therefore, detecting subpleural consolidation on ultrasound can effectively exclude a diagnosis of TTN [21,22,23].

**Figure 1:**
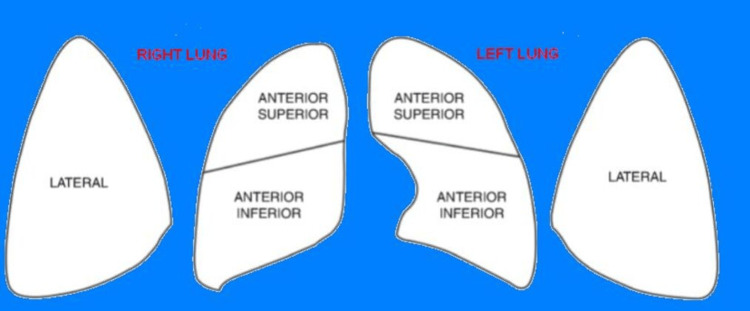
Description of the LUS score Each lung is divided into three areas, as shown in the figure LUS: lung ultrasound

LUS scores provide a standardized approach and allow for a semi-quantitative assessment of neonatal lung pathology and its progression. The neonatal LUS score is often calculated by dividing the anterior and lateral chest into six zones. Each lung is divided into three zones: anterior upper, anterior lower, and lateral. Each zone is scored from 0 to 3 points, resulting in a total score ranging from 0 to 18 points (Figure 1) [24,25].

This scoring system was first used in newborns in a 2015 prospective study led by French researcher Brat. The study divided the lung into six zones using a specific method and assigned a score from 0 to 3 for each zone based on the presence of A-lines, B-lines, and lung consolidation observed in the ultrasound. The total score could range from 0 to 18 points. This scoring system was used to quantitatively assess the effectiveness of exogenous surfactant administration in combination with continuous positive airway pressure (CPAP) in cases of NRDS. When the LUS score was above 2 or 4, the likelihood of requiring surfactant increased from 21% to 39% for neonates with a gestational age (GA) of 34 weeks or more, and from 25% to 72% for those less than 34 weeks. For newborns with a GA of less than 34 weeks, a score greater than 4 predicted the need for surfactant with 100% sensitivity.

Among the 130 neonates in this study, the LUS score significantly correlated with all oxygenation indices, regardless of GA. The score predicted the need for surfactant more accurately in preterm infants with a GA under 34 weeks [area under the receiver operating characteristic curve (AUC) = 0.93] compared to term and late-preterm infants with a GA of 34 weeks or more (AUC = 0.71). In infants with a GA under 34 weeks, an LUS score cutoff of 4 predicted surfactant administration with 100% sensitivity and 61% specificity, providing a post-test probability of 72% [25].

In a study by Copetti et al., LUS was performed on 40 neonates with clinical and radiological signs of RDS (mean GA of 27.2 ± 2.7 weeks and mean birth weight of 1,057 ± 361 g) and on 15 preterm infants without RDS (mean GA of 30.4 ± 3.4 weeks and mean birth weight of 1,775 ± 669 g). The study found that in all cases of RDS, LUS consistently revealed generalized alveolar-interstitial syndrome, also known as the "white lung" appearance. Other findings included pleural line abnormalities such as small subpleural consolidations, thickening, irregularity, and a coarse appearance, along with an absence of normal lung patterns ("spared areas"). The simultaneous presence of these ultrasound features allowed for the identification of RDS with a sensitivity and specificity of 100%. These findings suggest that LUS is a highly accurate diagnostic tool for RDS and could potentially be used as a screening method for early detection and timely administration of surfactants in preterm infants with respiratory distress [26].

A meta-analysis by Hiles et al. (2017) reviewed six studies comparing LUS with CXR for diagnosing NRDS. The analysis demonstrated a pooled sensitivity of 97% (95% CI: 0.94-0.99) and a specificity of 91% (95% CI: 0.86-0.95) for LUS in diagnosing NRDS, compared to CXR. However, the meta-analysis also revealed significant heterogeneity among the included studies, as indicated by an I² value >50% and χ² statistics, consistent with Cochrane Handbook recommendations (Higgins and Green, 2008). This suggests that subgroup analysis of prospective cohort studies may provide the most accurate assessment of test accuracy for LUS in NRDS [27,28]. In a study by Vergine et al. (2014), 59 neonates were included to evaluate the accuracy of LUS in diagnosing NRDS. The neonates had a mean GA of 33 ± 4 weeks and a mean birth weight of 2,145 ± 757 g. The study found that LUS demonstrated a sensitivity of 95.6% and a specificity of 94.4% for diagnosing NRDS, with a positive predictive value (PPV) of 91.6% and a negative predictive value (NPV) of 97.1%. These findings establish LUS as a highly precise and reliable diagnostic tool for identifying NRDS in newborns [29].

The Role of Lung Ultrasound in the Grading and Treatment of Neonatal Respiratory Distress Syndrome

Early identification and treatment of RDS are crucial in reducing the risk of death and bronchopulmonary dysplasia (BPD). Research indicates that infants who receive surfactant therapy within the first two to three hours of life have a lower risk of developing BPD and mortality compared to those who are treated later [30]. Recently introduced ultrasound features, such as the "ground-glass sign," indicating mild lung consolidation with a frosted glass-like appearance, and the "snowflake sign," showing lung consolidation with a distinct air bronchogram pattern resembling a snowflake, enhance the precision of diagnosing RDS. These signs not only help distinguish between different types of lung pathology but also provide a framework for assessing the severity of RDS, thereby improving both diagnostic accuracy and clinical management [31].

Liu et al. have proposed classifying RDS based on the severity of lung consolidation and the presence of major complications. RDS can be categorized into mild, moderate, and severe forms. In mild RDS, lung consolidation appears as a ground-glass sign on ultrasound, which may also be seen in the early or recovery stages of moderate to severe RDS. Moderate RDS is indicated by the snowflake sign on ultrasound, but not all lung areas are affected. Severe RDS is characterized by snowflake-type consolidation affecting all lung zones or by any degree of consolidation leading to severe complications, such as pulmonary hemorrhage, pneumothorax, massive atelectasis, or persistent pulmonary hypertension [32].

The recent study by Kartikeswar et al. (2022) investigated the use of LUS for diagnosing respiratory distress in neonates, using clinico-radiological diagnosis (clinical assessment plus CXR) as the reference standard. A secondary objective was to determine if a modified LUS score could predict the need for surfactant therapy. In this prospective observational study conducted over one year (January-December 2018) in a tertiary NICU, all preterm neonates with respiratory distress were evaluated with both LUS and CXR within the first two hours of admission. A total of 92 neonates were screened, and 61 were ultimately diagnosed with RDS. The Kappa statistic showed a moderate agreement of 0.639 between clinicoradiological diagnosis and LUS, and a strong agreement of 0.786 (95% CI: 0.678-0.983) between LUS and CXR diagnoses. The most frequent LUS finding in RDS was pleural line thickening (100%), followed by "white lung" (75.4%). The modified LUS score was significantly higher in infants requiring surfactant therapy [median (IQR): 49 (44, 53.5) vs. 29.5 (21, 46); p<0.0001]. The study concludes that LUS can predict the severity of RDS and the need for surfactant therapy, showing good concordance with clinical and radiologic diagnoses [33].

The study by Liu et al. (2022) evaluated the use of LUS to enhance the diagnosis and treatment of NRDS. Between March 2017 and 2018, 385 neonates in a NICU were assessed, of which 269 were diagnosed with RDS and received surfactant therapy (PS) based on LUS findings. The remaining 116 neonates did not meet the ultrasound criteria for RDS, did not receive PS, and achieved good clinical outcomes. The use of LUS reduced the misdiagnosis rate of RDS by 30.1% and decreased PS use by 30.1%. Among the 269 patients with RDS confirmed by LUS, 148 were treated with CUROSURF® at an average dose of 105.4 mg/kg, significantly lower than the recommended dose of 200 mg/kg. The study reported a 0% mortality rate, with no cases of ventilator-associated pneumonia or BPD. LUS can effectively reduce misdiagnosis rates and the need for surfactant use in RDS, ensuring better outcomes for neonates [34].

Between May and September 2012, a study was conducted to evaluate the effectiveness of LUS in diagnosing RDS in neonates compared to CXR. The study included 45 neonates with RDS and 30 neonates without any lung disease. The ultrasound identified characteristic signs of RDS, such as pulmonary consolidation, pleural line abnormalities, bilateral "white lung," and the absence of the A-line in 100% of RDS cases, while none of these signs were present in the control group. Pulmonary pulse was observed in 80% of the RDS cases. The results demonstrated that LUS had a sensitivity and specificity of 100% for diagnosing RDS, offering significant advantages such as the absence of ionizing radiation and the ability to be used repeatedly without risk. Therefore, LUS is a precise and safe method for diagnosing RDS in neonates and should be considered for use in neonatal units [35].

Wu et al. (2020) conducted a meta-analysis to assess the diagnostic performance of LUS for NRDS, comparing it to the conventional CXR. They compiled data from 10 studies, involving 887 neonates, to provide a robust evaluation of LUS. The pooled sensitivity of LUS was found to be 92% (95% CI: 89-94%), and the specificity was 95% (95% CI: 93-97%), indicating that LUS is highly effective at both detecting and excluding NRDS. The study also reported a positive likelihood ratio (PLR) of 20.23 (95% CI: 8.54-47.92), meaning that neonates with NRDS were over 20 times more likely to have a positive LUS result compared to those without the condition. Conversely, the negative likelihood ratio (NLR) was remarkably low at 0.07 (95% CI: 0.03-0.14), showing that a negative LUS result effectively rules out the presence of NRDS. The diagnostic odds ratio (DOR), which combines both sensitivity and specificity into a single measure of test effectiveness, was an impressive 455.30 (95% CI: 153.01-1354.79). The AUC was 0.9888, further supporting the high diagnostic accuracy of LUS [36].

This meta-analysis not only confirms the reliability of LUS in diagnosing NRDS but also highlights its superiority in terms of safety and convenience compared to CXR, particularly in reducing neonatal exposure to ionizing radiation. The study's results are consistent with earlier reviews, such as the one that analyzed data from six studies involving 480 neonates, which reported a pooled sensitivity of 97% and specificity of 91% for LUS. By including studies published from 1990 to 2019, Wu et al. provided a more extensive and updated analysis, reinforcing the clinical utility of LUS as a non-invasive and accurate diagnostic tool in NICUs [36].

Chest X-ray in respiratory distress syndrome

Most neonatal lung conditions are diagnosed using imaging methods. In managing these newborns admitted to NICUs, CXR plays an extremely important role in diagnosis and is also useful for assessing the positioning of tubes, probes, and catheters. Despite technological advances in imaging over the past decades and the increased use of LUS in some centers, CXRs continue to be widely used as an imaging method. Most newborns admitted to NICUs are premature and have a low birth weight, representing approximately 69% of admissions, with mortality most often attributed to respiratory complications [37].

Anatomically, children and newborns have significant differences in their airways and respiratory systems compared to adults, which leads to a predisposition to different pathologies. These differences are more evident at birth and in childhood. For example, a newborn's head is disproportionately larger than the neck and jaw, the tongue is large and positioned posteriorly, and there is physiological enlargement of the tonsils and adenoids. The trachea is shorter, and the glottic opening is larger than in adults. The cricoid cartilage is the narrowest part of the airway in children, whereas, in adults, the narrowest part of the upper airway is at the glottic opening. Additionally, compared to adults, the pulmonary alveoli in children have thicker walls at birth and are fewer in number [38].

For newborns, especially premature ones, CXRs should ideally be performed with a portable radiological device. The X-ray is performed in the anteroposterior view to provide sufficient diagnostic information and to reduce the radiation dose to patients. The first X-ray may also include abdominal imaging to assess the potential presence of intestinal air and to rule out any abdominal pathology that might cause respiratory symptoms. Radiology can also be used to evaluate the positions of intubation tubes or umbilical catheters [39]. Lateral decubitus X-rays can confirm a suspected pneumothorax or evaluate pleural effusion [40]. Radiologically, RDS is characterized by small lung volumes, horizontal ribs, a "ground glass" appearance, and air bronchograms. In severe cases, the appearance may resemble a "white lung." A normal CXR at six hours of life excludes the diagnosis of RDS. After administering exogenous surfactant, radiographic images show changes with the disappearance of granular opacities and an increase in lung volume [41].

Morris pointed out that the radiological features are closely related to the clinical severity of the condition, where atelectasis is represented by a bilateral fine granular or "ground-glass" appearance, which indicates the degree of lung opacity and reflects the spread of the disease. Additionally, depending on the stage of the disease, reduced lung expansion, dilated bronchioles, and air bronchograms can also be observed [42]. Children, especially those born prematurely, are particularly susceptible to the harmful effects of ionizing radiation due to their immature tissues and rapidly growing cells. The biological effects of medical radiation exposure depend on the radiosensitivity of the targeted tissues and organs [43], which is why pediatric radiological procedures, including X-rays, must follow the ALARA principle ("as low as reasonably achievable"), as recommended by the American College of Radiology and the International Atomic Energy Agency (IAEA) [44,45,46].

Silveira Neves et al. (2024) conducted a systematic review to evaluate the role of CXR in the diagnosis and severity assessment of NRDS, particularly in low-resource settings where access to diagnostic tools like CXR is limited. Despite the decreasing reliance on CXR due to the need for rapid clinical decisions, its use remains prevalent in managing NRDS. The review included 1,686 studies, out of which 23 involving 2,245 newborns were selected. All selected studies used CXR for diagnosing NRDS, with 21 (91%) using it to assess disease severity. Seven studies (30%) indicated that CXR is indispensable for diagnosing NRDS, while 10 studies (43%) found that alternative methods surpass CXR in various aspects, such as severity assessment, monitoring disease progression, predicting the need for surfactant therapy, anticipating CPAP failure, intubation needs, and aiding in differential diagnosis. The review concluded that CXR remains an important diagnostic tool for NRDS, although its continued use in scientific reports may not fully reflect current global clinical practices, especially in low-resource settings where early NRDS management remains a critical challenge for neonatal survival [47].

Advantages and disadvantages of the two imaging methods

For many years, bedside CXR has been considered the gold standard for diagnosing NRDS due to the inability to perform bedside CT. However, CXRs have several limitations:

Positioning Limitations

Bedside CXRs are typically performed in a supine position, which can obscure posterior lung lesions due to overlapping structures. This limitation is significant in NRDS patients who often require mechanical ventilation and experience ventilation/perfusion abnormalities in the posterior lung regions.

Time and Safety Concerns

Conducting a bedside CXR requires moving both the equipment and the critically ill infant, which can be time-consuming and risky. The process can lead to delays in diagnosis and may cause adverse events like temperature instability or displacement of medical devices such as tracheal tubes and oxygen masks.

Limited Dynamic Monitoring

CXRs do not provide real-time monitoring of a patient's condition, which is essential when there are sudden changes in a critically ill newborn's status. This limitation can hinder timely and accurate adjustments to the clinical management of the infant.

Radiation Risks

Frequent X-rays expose infants to ionizing radiation, increasing the risk of long-term health issues such as DNA damage and cancer, especially in vulnerable populations like preterm infants.

Given these challenges, certain alternative diagnostic methods have been explored. In 1990, Avni et al. first reported the use of LUS in diagnosing NRDS, suggesting that LUS could replace CXRs. Since then, neonatal LUS has gained widespread currency due to its high accuracy and specificity in diagnosing various neonatal lung conditions, including NRDS, TTN, meconium aspiration syndrome (MAS), neonatal pneumonia, and pneumothorax [48]. Between 1990 and 2006, studies investigated the cancer risk associated with X-ray exposure during fetal development and early childhood. According to Schulze-Rath et al. (2008), while no significant association was found between prenatal X-ray exposure and leukemia, the results regarding postnatal exposures were mixed. Some studies suggested a potential link between postnatal exposure and leukemia, as well as other cancers like solid tumors and brain tumors. However, these findings were influenced by limitations such as small sample sizes and imprecise exposure measurements, highlighting the need for further research [49].

LUS has several advantages over CXR, including real-time dynamic monitoring, no radiation exposure, and ease of use. In 2020, ESPNIC published international evidence-based guidelines that support the use of POCUS for critically ill neonates and children. These guidelines have further strengthened the role of LUS in NICUs, not only for diagnosing and differentiating NRDS but also for monitoring treatment effectiveness and guiding clinical decisions [50]. Federici et al. (2011) investigated the use of LUS to reduce the number of CXRs required for monitoring preterm newborns with very low birth weight (VLBW) diagnosed with RDS. Between April and September 2008, 21 newborns were assessed with LUS every 8-12 hours until clinical resolution of the disease was observed, and CXRs were only performed in unclear cases. A total of 105 ultrasounds were conducted, revealing "comet tail" artifacts and, in some cases, pulmonary consolidations. The average number of CXRs per patient during the study period was significantly lower than in the previous six months (2.6 ± 1 vs. 3.8 ± 1.5; p<0.05). The findings suggest that LUS can reduce the need for CXRs and radiation exposure, serving as an efficient alternative method for monitoring RDS in VLBW infants [51].

In another study, conducted by Liu et al. in 2014, the effectiveness of LUS in diagnosing RDS in neonates was evaluated. The study included 100 newborns between March 2012 and May 2013, divided into two groups: 50 with RDS and 50 as controls. LUS demonstrated a sensitivity and specificity of 100% for diagnosing RDS in the affected group, identifying key signs such as pulmonary consolidation, pleural line abnormalities, and bilateral "white lung." The study concluded that LUS is a reliable and accurate method for diagnosing RDS. It is non-invasive, free from ionizing radiation, cost-effective, easy to use, and can be performed at the bedside, making it ideal for use in NICUs [52].

Discussion

Diagnostic Accuracy and Reliability of Lung Ultrasound

LUS has shown a high sensitivity and specificity in diagnosing NRDS, as demonstrated in multiple studies. For instance, a meta-analysis reported a pooled sensitivity of 92% and specificity of 95% for LUS, which are comparable to or better than those of CXR. This high diagnostic accuracy makes LUS a reliable tool for early detection and management of NRDS, particularly in settings where rapid decision-making is crucial.

Reduction of Radiation Exposure

One of the primary advantages of LUS over CXR is the elimination of ionizing radiation, which is particularly important in neonatology due to the increased vulnerability of neonates to radiation-related risks. Studies have highlighted that using LUS can significantly reduce the cumulative radiation dose received by neonates, thereby potentially lowering the long-term risks associated with repeated exposure to X-rays, such as DNA damage and cancer.

Real-Time Monitoring and Bedside Availability

LUS allows for real-time monitoring and can be performed at the bedside, making it a dynamic tool for continuous assessment of lung conditions. This capability is especially beneficial in NICUs, where the ability to make quick, informed decisions is vital. The use of LUS can also help guide interventions like surfactant administration more effectively than CXR, which does not provide real-time feedback.

*Challenges and Limitations* 

Despite the many advantages, the widespread adoption of LUS is limited by several factors, including the need for specialized training and experience to interpret ultrasound findings accurately. There is also a lack of standardized protocols and educational programs for LUS in neonatal care, which can hinder its effective implementation across different healthcare settings.

Comparative Effectiveness in Different Clinical Scenarios

While CXR has been traditionally used to diagnose and monitor NRDS and other respiratory conditions in neonates, LUS has shown potential in not only providing a diagnosis but also assessing the severity of conditions like NRDS and TTN. Studies have shown that LUS can differentiate between these conditions effectively, which is crucial for ensuring appropriate management.

Need for Further Research

Further studies are required to refine LUS protocols and validate its use across diverse clinical settings. Research should also focus on optimizing training programs for healthcare professionals and assessing the cost-effectiveness of integrating LUS into standard neonatal care practices. Additionally, long-term studies are needed to evaluate the outcomes of neonates diagnosed and managed with LUS compared to those managed with traditional imaging modalities like CXR.

## Conclusions

LUS should be included as a complementary method to CXR in the diagnostic algorithms for NRDS. The introduction of LUS as a non-hazardous imaging method could reduce the number of X-ray examinations, replacing some of them with ultrasound and thus decreasing the ionizing radiation exposure in neonates. Additionally, LUS is a cost-effective method compared to CXRs, as it does not require expensive equipment or consumables like ionizing radiation devices do. The average cost of an ultrasound is significantly lower than that of repeated CXRs, making it not only a safer but also a more economical option, especially in NICUs, where frequent imaging is required. Ultrasound is a highly accurate and accessible imaging method with virtually no contraindications and avoids the risks associated with ionizing radiation exposure. Hence, its use as a diagnostic tool has significantly increased in recent years. However, the use of ultrasound in investigating chest diseases in neonates remains quite limited.

The guidelines from ESPNIC recognize the importance of using POCUS in the care of critically ill neonates. Nevertheless, the widespread use of LUS is hindered by the lack of standardized educational programs and the implementation of effective quality assurance measures. Studies continue to highlight the need to reduce ionizing radiation exposure in clinical practice and to promote the use of LUS as a complementary method for diagnosing and monitoring NRDS in neonates. We conclude that LUS has the potential to improve the safety and quality of care in NICUs, offering an effective and non-invasive alternative for the diagnosis and management of NRDS.
